# Extrarenal Visceral Arteries Injuries during Left Radical Nephrectomy: A 50-Year Continuing Problem

**DOI:** 10.3390/jcm13206125

**Published:** 2024-10-14

**Authors:** Marco Catarci, Leonardo Antonio Montemurro, Michele Benedetti, Paolo Ciano, Massimiliano Millarelli, Roberto Chiappa

**Affiliations:** 1 General Surgery Unit, Sandro Pertini Hospital, ASL Roma 2, Via dei Monti Tiburtini 385, 00157 Rome, Italy; leonardo.montemurro@aslroma2.it (L.A.M.); michele.benedetti@aslroma2.it (M.B.); paolo.ciano@aslroma2.it (P.C.); 2 Vascular Surgery Unit, Sandro Pertini Hospital, ASL Roma 2, Via dei Monti Tiburtini 385, 00157 Rome, Italy; massimiliano.millarelli@aslroma2.it (M.M.); roberto.chiappa@aslroma2.it (R.C.)

**Keywords:** superior mesenteric artery injuries, celiac trunk injuries, left radical nephrectomy

## Abstract

Due to their proximity to the left renal hilum, injuries to the superior mesenteric artery and celiac trunk are still reported during left radical nephrectomy, whether performed via open, laparoscopic, or robotic methods. The aim of this 50-year narrative review is to emphasize the anatomical and pathophysiological bases, risk factors, and strategies for the prevention, diagnosis, and treatment of such injuries.

## 1. Introduction

Nephrectomy is a surgical procedure that involves kidney removal. It can be performed for various reasons including renal tumors, chronic kidney disease, and severe kidney injury. Left radical nephrectomy (i.e., complete removal of the left kidney along with surrounding tissues, including the adrenal gland and nearby lymph nodes) is generally performed for the treatment of left-sided renal cell carcinoma, particularly in cases of large tumors or involvement of the surrounding structures. Despite many advancements that have enhanced the precision and efficacy of this surgery (laparoscopic and robotic approaches), allowing for minimally invasive procedures with reduced recovery times and complication rates, several extrarenal visceral artery injuries have been reported since their first descriptions 50 years ago [1,2]. Understanding visceral artery injuries during left radical nephrectomy is crucial for several reasons. First, these injuries can lead to significant morbidity and mortality, thereby affecting patient outcomes. Second, the visceral arteries involved, including the superior mesenteric artery (SMA) and celiac trunk (CT), play vital roles in supplying blood to the stomach, duodenum, small bowel, proximal large bowel, liver, pancreas, and spleen. Injury to these vessels can result in ischemia, organ dysfunction, and death. Therefore, a comprehensive understanding of the anatomy, mechanisms of injury, clinical implications, and prevention strategies is essential for surgeons performing nephrectomy. This article aims to provide an in-depth exploration of extrarenal visceral artery injuries during left radical nephrectomy. It covers the anatomy of the renal vasculature, the mechanisms of injury, the clinical implications, the prevention strategies, and a review of relevant case studies. By synthesizing this information, this article seeks to enhance the knowledge of this critical aspect of nephrectomy and to improve surgical outcomes.

## 2. Renal and Visceral Vascular Anatomy

### 2.1. Renal Arteries (RAs)

The renal arteries (RAs) are the primary blood supply to the kidneys, arising from the lateral aspect of the abdominal aorta, typically at the level of the first or second lumbar vertebra, just below the origin of the SMA [3]. The RAs supply a large amount of blood to the kidneys, accounting for nearly one-third of the cardiac output. The number of RAs may vary among individuals, with double or triple RAs in up to one-third of the cases [4]. Because of the mutual location of the aorta, inferior vena cava, and kidneys in the abdominal cavity, the right renal artery (RRA) is normally longer than the left renal artery (LRA). The RRA originates from the right lateral margin of the aorta, with which it forms an angle just over 60°, and passes behind the inferior vena cava, right renal vein, head of the pancreas, and descending portion of the duodenum, describing a curved trajectory with a posterior concavity against the psoas major muscle. The overall length of the RRA is approximately 7 cm. The LRA originates from the left lateral margin of the aorta, with which it forms an almost right angle, in a slightly more cranial position than the right one; it runs behind the left renal vein, body of the pancreas, and splenic vein and crosses the inferior mesenteric vein. It is slightly shorter than the RRA, with a total length of approximately 5 cm. Each renal artery divides into anterior and posterior branches, which further branch into segmental arteries that supply specific regions of the kidney. Understanding this vascular anatomy is essential to avoid inadvertent RA injuries during nephrectomy.

### 2.2. Superior Mesenteric Artery (SMA)

The superior mesenteric artery (SMA) is a large arterial branch that arises from the aorta at the level of the first lumbar vertebra, immediately below the celiac trunk. It ensures blood supply to a large part of the intestine, from the last portion of the duodenum to the middle of the transverse colon. Approximately 24/25 cm long, it runs behind the pancreas and then heads forward until it bypasses the third portion of the duodenum to reach the mesentery. Important branches arise along its path. It ends by anastomosing with the ileal branch of the ileocolic artery and with the last jejunal collateral. It is responsible for the blood supply of the duodenum–pancreas (together with the celiac trunk), small intestine, and colon up to the distal transversus, where the anastomotic arch of Riolano is located between the left branch of the middle colic artery and ascending branch of the left colic artery (branch of the inferior mesenteric artery). From its origin, the SMA is directed inferiorly, approximately 3 cm, along the ventral wall of the aorta, behind the isthmus of the pancreas, and is then directed at an acute angle away from the aorta on the lower margin of the pancreas. It descends diagonally from the uncinate process and the third portion of the duodenum, which crosses vertically. It then penetrates the root of the mesentery, which it follows for a few centimeters, and is inserted into the floating part of the mesentery, where it ends.

### 2.3. Celiac Trunk (CT)

The celiac trunk, celiac artery, or also celiac tripod of Haller (CT) is a voluminous arterial branch that arises from the abdominal aorta at the level of the twelfth thoracic vertebra. After about two or three centimeters from the origin, three arterial branches arise from the trunk (hence the name “tripod”): the left gastric artery; common hepatic or gastrohepatic artery; and lienal or splenic artery. It is responsible for supplying blood to the liver, distal esophagus, stomach, duodenum, pancreas, and spleen.

### 2.4. Spatial Relationships

**Figure 1:**
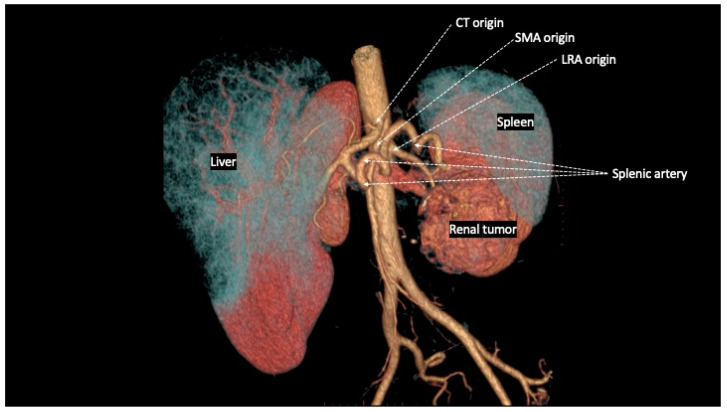
Three-dimensional reconstruction of vascular anatomy spatial relationships on computed tomography with intravenous contrast: the patient had combined SMA and splenic artery injuries during laparoscopic left radical nephrectomy for a large renal cell carcinoma with periaortic tissue involvement.

The spatial relationships between the CT, SMA, and RA are extremely variable under physiological conditions and are clearly influenced by different pathological conditions. Regarding the common origin from the aorta, both the CT and SMA arise from the anterior wall in most cases, whereas RAs originate from the lateral or anterolateral wall of the aorta. In particular, the LRA originates from the anterolateral wall in 52%, from the lateral wall in 45%, and from the posterior wall of the aorta in 3% of cases [5]. Cadaveric studies have shown great variability in the distance between the ostia of origin of the CT, SMA, and RA, generally not exceeding the value of 10 mm [5,6,7,8], varying around an average of 6 mm between the SMA and the LRA [9,10,11] in studies based on in vivo computed tomography scans (Figure 1).

Moreover, the left renal vein (LRV) generally crosses the abdominal aorta anteriorly and courses posterior to the SMA at the fork of the angle between the SMA and aorta. Therefore, the course of the LRA occurs posterior to the LRV, except in 10% of cases in which the LRV has a retroaortic course [12]. Finally, there are several collateral pathways between the visceral arteries that may compensate for the effect of ischemia derived from their injuries. The CT presents collateral circles with the SMA at the level of the superior and inferior pancreaticoduodenal arches. The SMA is in communication with the inferior mesenteric artery (IMA) through the arch of Riolano and the marginal artery of Drummond [13].

## 3. Extrarenal Visceral Arterial Injuries during Left Nephrectomy

### 3.1. Literature Review

Injuries to the SMA and/or CT during left nephrectomy likely occur more often than the small number of cases reported to date in the literature [14,15]. The highest incidence was reported by Ritchey in 1992, in which a lesion of the SMA occurred in approximately 2% of children undergoing open nephrectomy for Wilms tumor with venous involvement [16] and in 1.6% of adults undergoing laparoscopic nephrectomy for cancer [17]. There have been several other sporadic cases of SMA and/or CT injuries during left nephrectomy, either through open (both laparotomic or retroperitoneal), laparoscopic, or robotic approaches. Twenty-five cases of iatrogenic extrarenal visceral arteries injuries have been reported to date (Table 1), mostly (80%), with favorable outcomes after revascularization.

Therefore, it is probable that, in addition to an “under reporting” phenomenon common to all iatrogenic injuries, there is also a “publication bias”, or the natural tendency to publish cases with a favorable outcome and not to publicize those with an unfavorable outcome. Vascular complications, including those specific to the renal vessels, are the most frequently encountered during nephrectomy [14], and the mortality rate resulting from any traumatic lesion of the proximal tract of the SMA reaches 75% [32,33]. Despite the fact that early identification of the lesion followed by immediate revascularization is often linked to a positive result, there are still instances where delayed recognition, without revascularization of the SMA and/or CT, can lead to a favorable outcome [19,27,29] due to the development of enough collateral circulation to ensure proper visceral perfusion, as seen in post-traumatic injuries as well [34].

### 3.2. Risk Factors

**Figure 2:**
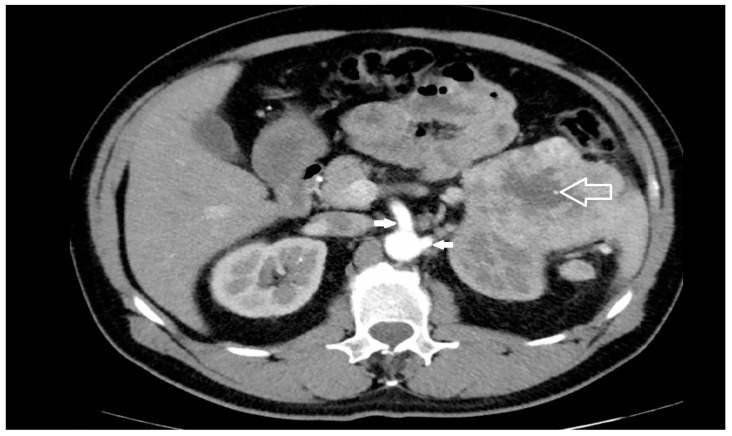
Large renal cell cancer of the left upper kidney (empty arrow): close proximity of the origin of SMA and LRA (solid arrows) from the aorta is evident.

The risk factors most commonly reported for the occurrence of an iatrogenic injury of the SMA and/or CT during left nephrectomy/adrenalectomy are the close spatial relationship between renal and visceral arteries [5,6,7,8,9,10,11]; surgery indicated for large neoplasms of the left upper renal pole or left adrenal gland (Figure 2), extra-renal spread, or bulky hilar lymph node involvement [15,21]; surgery indicated for inflammatory renal diseases or completion nephrectomy after partial resection with perivisceral inflammatory adhesion to the aorta and its visceral branches [15]; morbid obesity [35]; and the surgeon’s lack of experience [28,36].

### 3.3. Pathogenesis, Prevention and Diagnosis

**Figure 3:**
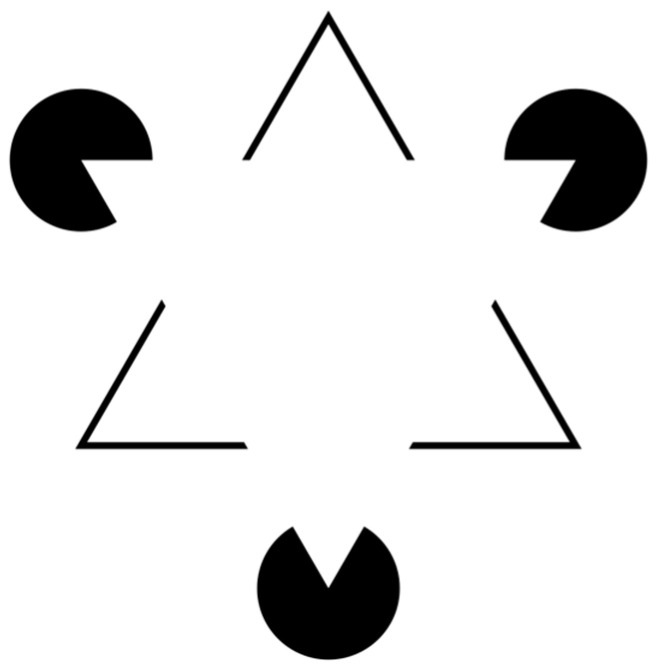
The Kanizsa triangle [38].

In the vast majority of cases, the pathogenesis of these lesions depends on a perceptive deficit of the surgeon who, convinced that they have identified the left renal artery to be divided, finds themselves dealing with the SMA and/or the CT [15,30]. This pathogenetic mechanism is comparable to that observed in the majority of “classic” iatrogenic injuries of the main bile duct during cholecystectomy [37]. The Kanizsa triangle (Figure 3) is an optical illusion, which was first described in 1955 by Italian psychologist Gaetano Kanizsa [38], which gives a perfect idea of the perceptive deficit that leads the surgeon into error.

In Figure 3, we can “see” two white equilateral triangles, one superimposed on the other, even if neither triangle is actually drawn. This effect is known as subjective or illusory profiling. Furthermore, the nonexistent white triangle appears to be brighter than the surrounding area, while that area has the same brightness as the adjacent areas. This phenomenon occurs because our perceptive apparatus has an innate organizational tendency constituted by figure/ground articulation, according to which there is no figure without a background; this also happens with figures obtained with physically non-existent margins such as this triangle. This is because our perceptual evaluation requires figure/background contrast, and even when this is not present, the same is created.

**Figure 4:**
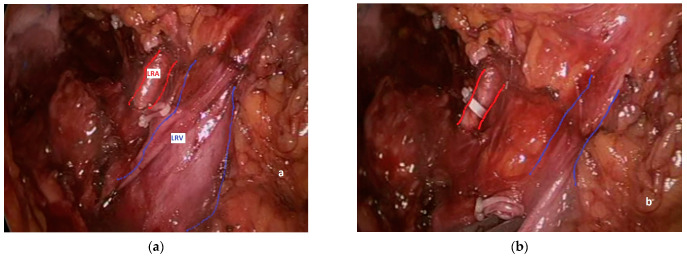
Dissection of the left renal hilum during left nephrectomy: (**a**) the LRA (red lines) lies posteriorly to a blood-filled LRV (blue lines); (**b**) once the LRA (red lines) is clipped, the LRV (blue lines) appears emptied.

During left nephrectomy, the perceptive deficit manifests itself in a completely similar way: the surgeon determines a lesion of the SMA and/or the CT convinced that he is instead dealing with one or more LRA [15,30]. Actually, during transabdominal left nephrectomy, either open or minimally invasive, the surgeon generally takes down the splenic flexure of the colon and directly dissects the renal hilum, finding the LRV first and the LRA immediately after (Figure 4a), lying behind it in 90% of the cases [12]. Thereafter, an occluding clip is generally first placed on the LRA (Figure 4b), followed by clipping and division of the left renal vein, to avoid renal venous engorgement.

If a single LRA is identified on preoperative imaging studies and engorgement of the LRV is noticed after LRA clip placement, the possibility that the vessel just clipped is not the LRA could arise before vessel division. This is the first moment during nephrectomy, in which the surgeon may feel a reasonable suspicion that his perception of the anatomic setting is incorrect. Further periaortic dissection and identification of the true LRA may allow the removal of the previously placed clip and proceed to nephrectomy. This kind of injury, when promptly recognized and treated, has little or no consequences on the patient’s outcomes, but it is very rare. Only one similar case has been reported to date [30]. Unfortunately, in the vast majority of cases, the procedure continues after clipping and division of the misperceived LRA, and further intraoperative suspicion of extrarenal arterial injury may arise because of the following: (1) venous engorgement of the renal stump of the divided left renal vein; (2) identification of an LRA anterior to the LRV; (3) atypical course of the artery (i.e., transversal); (4) arterial origin from the anterior aortic wall; (5) finding another artery after division of the first LRA (with preoperative imaging negative for multiple LRA); and (6) impossibility of clear identification of the left aortic wall due to the disease (neoplastic and/or inflammatory). Although anatomic misperception is the main pathogenetic mechanism of such injuries, excessive bleeding and poor exposure of the operative field are frequently reported as concurrent factors, possibly linked to the surgeon’s lack of experience [15,21,28,30].

**Figure 5:**
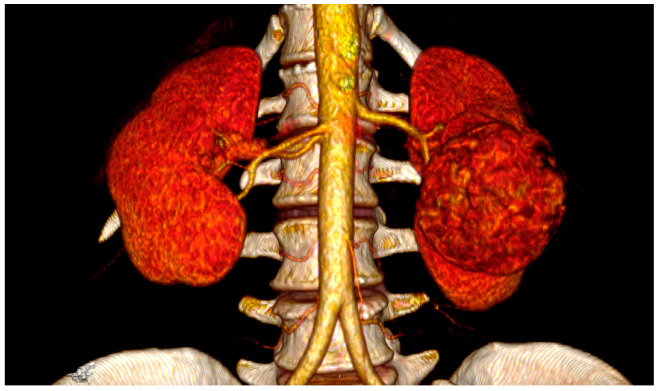
Three-dimensional reconstruction of renal vascular anatomy on axial computed tomography scan with intravenous contrast: a single LRA and double RRA are easily recognizable.

Considering the above-reported variability in the origin of the RA close to the visceral vessels, the first measure for the prevention of iatrogenic arterial injuries is preoperative evaluation of the vascular anatomy of the kidneys before nephrectomy [22,39] using a computed tomography scan with intravenous contrast (Figure 5), which allows the number and course of renal arteries to be identified in 99% of cases [11,40].

Recently, there has been a rise in the usage of three-dimensional reconstructions of vascular anatomy derived from CT and MRI images [41,42,43]. While primarily focused on improving partial nephrectomy, its application in left nephrectomy for large renal tumors (Figure 5) is unquestionably beneficial. It helps assess spatial relationships between the renal artery and visceral arteries (Figure 1), serving as a valuable tool in preventing accidental injuries. Simultaneously, virtual and augmented reality are creating new opportunities in medical practice [44]. Their utilization was also documented in kidney surgery [45,46,47,48], showing a beneficial impact on results in randomized trials [46]. In the near future, it seems quite clear that utilizing virtual reality for surgical planning before the surgery and displaying vascular anatomy through augmented reality during the operation will soon be effective methods for avoiding such injuries. The emergence of a new line of medical headsets for enhanced virtual and augmented reality in urology offers fresh opportunities for innovation, making it easier for all to access. These groundbreaking headsets provide an immersive experience without the need for a controller, allowing for the integration of preoperative planning and intraoperative navigation, enhancing spatial awareness [49].

However, in a significant number of instances, the diagnosis may not be initially suspected until after the nephrectomy, when the color of the small bowel changes due to reduced blood flow, or later during the recovery period with symptoms such as abdominal pain, metabolic acidosis, and elevated serum lactates indicating intestinal necrosis.

### 3.4. Clinical Consequences and Treatment

The outcomes of extrarenal visceral artery injuries during nephrectomy vary based on three factors: (1) the anatomical localization of the lesion and presence of collateral circulation, generally more developed in patients with atherosclerotic stenosis of the SMA; (2) the timing of diagnosis and repair of the lesion using mesenteric revascularization techniques; and (3) the individual response to the systemic inflammatory response syndrome caused by tissue damage from ischemia/reperfusion and sepsis. Typically, the outcome for these patients is primarily conditioned by the development of splanchnic ischemia, resulting in organ damage (intestinal, hepatic, and pancreatic necrosis) and systemic damage (ischemia/reperfusion syndrome, systemic inflammatory response syndrome). The main cause of death is often the progression of sepsis due to bacterial translocation or peritonitis from acute intestinal necrosis, leading to multi-organ failure.

The severity of intestinal ischemia and mortality related to SMA injury is mainly determined by its anatomical location, with zone 1 lesions (between the aortic origin of the SMA and the emergence of the inferior pancreaticoduodenal artery) being the most common during nephrectomy, resulting in a mortality rate reported for traumatic lesions [32,33] of over 75% of cases. This occurs when the collateral circulation with the CT through the upper pancreaticoduodenal arches (branch of the CT through the hepatic and gastroduodenal artery) and the lower pancreaticoduodenal arches (branch of the SMA) is compromised, leading to residual perfusion of the SMA territory only through the existing collateral circles with the inferior mesenteric artery (arcade of Riolano and marginal arcade of Drummond). Due to the same cause, concomitant damage to the SMA and CT may result in increased mortality rates.

When the lesion is diagnosed early during surgery, it is recommended to involve a vascular surgery specialist promptly for urgent revascularization of the SMA, CT, or both using various procedures like direct anastomosis or aortic reimplantation, autologous vein or prosthetic interposition graft, or bypass with the splenic artery [18,20,23,24,25,30]. A 6 h diagnostic delay results in doubling of mortality rates [50]. However, even if the lesion is diagnosed later in the postoperative period after intestinal ischemia, necrosis, and peritonitis have occurred, it is still recommended to combine revascularization with the necessary resection of the ischemic intestinal tract [51], as patients who undergo revascularization have a significantly lower mortality rate compared to those who do not (40% vs. 60% [52]).

The variability in individual response to ischemia–reperfusion injury is very high. Ischemia ultimately causes tissue damage and cell death due to a lack of oxygen and nutrients. Extensive cellular demise may lead to ulceration and perforation of the intestinal wall, further advancing towards septic shock, despite the fact that the initial cause of sepsis is associated with bacterial translocation, starting with mucosal necrosis. Reperfusion of ischemic and injured tissue, even before surgical revascularization via slowed flow through collateral circulation, leads to increased oxygen influx. This results in the production of reactive oxygen and nitrogen species that interact with other molecules, causing oxidative stress like lipid peroxidation, protein carbonylation, and DNA oxidation, leading to additional cell damage and death [53]. The death of tissue leads to the production of proinflammatory cytokines and chemokines like tumor necrosis factor-α and interleukin-6, causing a series of effects such as heightened vascular permeability, bacterial migration, and activation and attachment of neutrophils. Inflammatory response syndrome (SIRS) is worsened by proinflammatory mediators discharged by mast cells and macrophages. Despite more than three decades of research in both experimental and clinical settings, there is still no successful treatment that can stop the cascade from SIRS to septic shock to multiple organ failure to death [54].

## 4. Limitations

Even after conducting a thorough literature review, which included prominent search engines, reference lists, and gray literature, the authors acknowledge the possibility of overlooking additional instances of iatrogenic injuries to extrarenal visceral arteries. Additionally, due to the lack of prospective studies on this particular subject, the majority of this narrative review relied on case reports or small series.

## 5. Conclusions

Iatrogenic lesions of the visceral arteries during left radical nephrectomy have been consistently reported during left radical nephrectomy for the past five decades, representing a significant surgical challenge with potentially devastating consequences. Understanding the anatomy of the renal and visceral vasculature, the mechanisms of injury, and management strategies is crucial for improving patient outcomes. Prevention, prompt recognition, and intervention are essential to mitigate the risks associated with these injuries.

## Figures and Tables

**Table 1:** SMA and/or CT injuries during left nephrectomy.

Author, Year (Ref.)	Approach	No. of Cases	Artery	Diagnosis	Treatment	Outcome
Roseau, 1973 [1]	Open	1	SMA	Intraop.	Revasc.	Alive
Arvis, 1974 [2]	Open	1	SMA	8 days	Revasc.	Dead
Court, 1976 [18]	Open	1	SMA	Intraop.	Revasc.	Alive
Bhanot, 1980 [19]	Open	2	SMA	Intraop.	Conserv.	Alive
Moul, 1991 [20]	Open	1	SMA/CT	Intraop.	Revasc.	Dead
Ritchey, 1992 [16]	Open	4	SMA	Intraop.	Revasc.	Alive
	Open	1	SMA	24 h	Conserv.	Dead
Siqueira, 2002 [17]	Lap	1	SMA	Intraop.	Revasc.	Dead
Blunt, 2004 [20]	Open	1	SMA	Intraop.	Revasc.	Alive
Kumar, 2007 [21]	Open	1	SMA	Intraop.	Revasc.	Alive
Nevoux, 2008 [22]	Open	1	SMA/CT	Intraop.	Revasc.	Alive
	Lap	1	SMA/CT	48 h	Revasc.	Dead
Abu-Ghazala, 2010 [23]	Lap	1	SMA/CT	Intraop.	Revasc.	Alive
Afonso, 2019 [24]	Open	1	SMA	Intraop.	Revasc.	Alive
Kumar, 2019 [25]	Robotic	1	SMA	Intraop.	Revasc.	Alive
Zhang, 2020 [26]	Open	1	SMA	Intraop.	Revasc.	Alive
Deb, 2021 [27]	Open	1	SMA	12 h	Revasc.	Alive
Mayor, 2022 [28]	Robotic	1	SMA	Intraop.	Revasc.	Alive
Ashraf, 2022 [29]	Retroper	1	SMA	24 h	Conserv.	Alive
Sayegh, 2023 [30]	Robotic	1	SMA	Intraop.	Revasc.	Alive
Huang, 2024 [31]	Open	1	SMA	Intraop.	Revasc.	Alive

Intraop.: intraoperative; Revasc.: revascularization; SMA: superior mesenteric artery; CT: celiac trunk; Conserv.: conservative (non-operative) treatment; Lap.: laparoscopic; Retroper.: retroperitoneal.

## Data Availability

Not applicable.
